# Antibiotic prescribing in mental health units across the Veterans’ Health Administration: How much and how appropriate?

**DOI:** 10.1017/ice.2021.432

**Published:** 2023-02

**Authors:** Jay J. Tieri, Bruce Alexander, Jason A. Egge, Brett H. Heintz, Daniel J. Livorsi

**Affiliations:** 1 Iowa City VA Health Care System, Iowa City, Iowa; 2 Division of Infectious Diseases, Carver College of Medicine, University of Iowa, Iowa City, Iowa

## Abstract

We evaluated antibiotic-prescribing across 111 mental health units in the Veterans’ Health Administration. We found that accurate diagnosis of urinary tract infections is a major area for improvement. Because non–mental-health clinicians were involved in most antibiotic-prescribing decisions, stewardship interventions for mental health patients should have a broad target audience to be effective.

Even though all US hospitals are required to have an antibiotic stewardship program, standard stewardship metrics like the standardized antimicrobial administration ratio do not capture antibiotic use in a hospital’s mental health units.^1^ It is unclear how frequently antibiotics are used in these settings and whether there are opportunities to improve antibiotic-prescribing.

The objective of this study was to describe and evaluate antibiotic use across inpatient mental health units in an integrated healthcare network known as the Veterans’ Health Administration (VHA).

## Methods

The Institutional Review Board of the University of Iowa and the Research & Development Committee of the Iowa City Veterans’ Affairs Health Care System approved this study and waived informed consent.

Using the Veterans’ Affairs Informatics and Computing Infrastructure (VINCI), we collected administrative data on all patients admitted to an inpatient mental health ward in VHA between January 1, 2016 and December 31, 2018. This cohort included data from inpatient mental health units across 111 unique medical centers, including 105 medical centers with an acute-care hospital. We excluded substance abuse rehabilitation units and domiciliaries.

Inpatient and postdischarge antibiotics were collected from the bar-coded medication administration records (BCMA) and outpatient medication files, respectively. Antibiotics included all agents and routes of administration listed in the National Healthcare Safety Network’s (NHSN) Antimicrobial Use protocol.^1^ For each unique facility, antibiotic days of therapy (DOT) and days present were aggregated based on NHSN methodology.

Manual chart reviews were performed in a subset of the above cohort to assess adherence to standard recommended practices and professional guidelines for cystitis, or urinary tract infections (UTI), skin and soft-tissue infections (SSTIs), and acute respiratory tract infections (ARIs). Admissions with a qualifying ICD-10 code (Supplementary Table 1 online) were eligible for inclusion. For each infection type, we randomly selected an eligible admission from each facility. If a patient admission met our exclusion criteria (Supplementary Table 2 online), we continued to randomly sample eligible admissions from that facility until a case could be fully adjudicated using our criteria. At some sites a qualifying case could not be found. For each case adjudication, assessments were based on established algorithms (Supplementary Figs. 1–7).^2,3^ The primary review was performed by a postgraduate year 1 pharmacy resident (J.T.). An infectious disease (ID)–trained clinician (D.L. or B.H.) performed a secondary review of all included cases. We monitored the number of cases in which the ID-trained clinician changed the assessment.

**Table 1.** Characteristics of 252,588 Patient Admissions to Mental Health Units in the Veterans’ Health Administration, 2016–2018

Characteristic	Antibiotic Received (n = 27,401)	No antibiotic Received (n = 225,187)	Total (n = 252,588)
Age, mean y (SD)	54.7 (14.2)	50.8 (14.3)	51.2 (14.4)
**Sex, no. (%)**			
Male	23,168 (84.5)	204,307 (90.7)	227,475 (90.1)
Female	4,233 (15.5)	20,880 (9.3)	25,113 (9.9)
**Race, no. (%)**			
White	17,863 (65.2)	145,126 (64.4)	162,989 (64.5)
Black or African American	7,646 (27.9)	63,225 (28.1)	70,871 (28.0)
American Indian or Alaska Native	272 (1.0)	2,002 (0.9)	2,274 (0.9)
Native Hawaiian or Pacific Islander	173 (0.6)	1,469 (0.7)	1,642 (0.7)
Asian	115 (0.4)	1,404 (0.6)	1,519 (0.6)
Missing	1,332 (4.9)	11,961 (5.3)	13,293 (5.3)
**Ethnicity, no. (%)**			
Hispanic or Latino	1,295 (4.7)	13,623 (6.0)	14,918 (5.9)
**Psychiatric diagnosis, no. (%)**			
Substance use disorder	19,920 (72.7)	173,123 (67.8)	193,043 (76.4)
Schizoaffective disorder	17,284 (63.1)	143,330 (63.6)	160,614 (63.6)
Anxiety disorder	14,545 (53.1)	128,240 (56.9)	142,785 (56.5)
Psychosis	6,903 (25.2)	47,313 (21.0)	54,216 (21.5)
Personality disorder	4,224 (15.4)	33,103 (14.7)	37,327 (14.8)
**Comorbid conditions, no. (%)**			
Drug abuse	14,682 (53.6)	121,648 (54.0)	136,330 (54.0)
Alcohol use disorder	13,434 (49.0)	120,291 (53.4)	133,725 (52.9)
Asthma/COPD	6,478 (23.6)	38,507 (17.1)	44,985 (17.8)
Diabetes	6,299 (23.0)	38,279 (17.0)	44,578 (17.6)
Neurologic disorder	3,544 (12.9)	19,294 (8.6)	22,838 (9.0)
Dementia	2,773 (10.1)	11,551 (5.1)	14,324 (5.7)
Renal failure	2,044 (7.5)	10,339 (4.6)	12,383 (4.9)
**Length of stay, median d (IQR)**	7 (3–13)	5 (3–9)	5 (3–9)

**Table 2.** Assessment of Guideline-Concordant Diagnoses and Antibiotic Management for Inpatient Mental Health Patients Diagnosed With Urinary Tract Infections, Skin and Soft-Tissue Infections, and Acute Respiratory Tract Infections Across 111 VHA Medical Centers, 2016–2018

Variable	Urinary Tract Infections (n = 111)	Skin and Soft-Tissue Infections (n = 101)	Acute Respiratory Tract Infections (n = 108)
Documentation and laboratory findings, if applicable, supported diagnosis of infection			
Yes	27 (24.3)	95 (94.1)^c^	108 (100)
No	63 (56.8)^a^	6 (5.9)^d^	0 (0)
Unable to assess	21 (18.9)^b^	…	…
Guideline-concordant antibiotic selection, no (%)^e^			
Yes	38 (79.2)	75 (78.9)	79 (73.1)^f^
No	10 (20.8)	20 (21.1)	29 (26.9)
Guideline concordant-antibiotic duration, no. (%)^e^,^g^			
Concordant	29 (60.4)	86 (90.5)	79 (73.1)^f^
Too long	18 (37.5)	9 (9.5)	29 (26.9)^h^
Too short	1 (2.1)	0	0
Antibiotic duration, median d (IQR)^e^	7 (6.5–10)	8 (6–10)	0 (0–5)^i^
Secondary reviewer changed original assessment, no (%)	12 (10.8)	36 (35.6)	2 (1.9)

Note. IQR, interquartile range.

a There were 56 cases with the documented absence of local genitourinary symptoms (ie, asymptomatic) and an additional 7 cases had urine cultures collected that were negative before antibiotic therapy.

b In these 21 cases, documentation in the medical record did not specify whether or not the patient had local genitourinary symptoms, such as dysuria, urgency, frequency, etc.

c There were 39 purulent infections and 56 nonpurulent infections. Of the purulent infections, 21 underwent incision and drainage.

d In these 6 cases, the documentation of skin findings was not suggestive of cellulitis. These were all cases with bilateral lower leg skin changes that were thought to be chronic.

e This category only includes cases in which documentation and laboratory findings, if applicable, supported the diagnosis of infection. For the diagnosis of urinary tract infections, cases deemed “unable to assess” were also included. Antibiotic selection was assessed on day 3 of therapy.

f In these 79 cases, an antibiotic was both indicated and prescribed in 3 cases, and in 76 cases an antibiotic was not indicated and was not prescribed.

g To assess guideline-concordant duration, any duration of therapy that was ≤ or ≥2 days from the guideline recommendation was considered concordant (Supplementary Figs. 1–7 online).

h In these 29 patients who did not meet criteria for antibiotic therapy, the median duration of antibiotics was 5 days (IQR, 5–10).

i In the 32 patients with an ARI who were prescribed an antibiotic, the median duration of therapy was 5 days (IQR, 5–10).

## Results

### Antibiotic use

There were 252,588 patient admissions across the 111 mental health units. Mental health units averaged 759 admissions per year (SD, 436). During the study period, 27,401 (10.9%) patient admissions had antibiotics administered while hospitalized on the mental health unit (Table 1). The diagnoses that were most commonly associated with an antibiotic prescription were UTI (n = 5,026, 18.3%), SSTI (n = 2,992, 10.9%), and ARIs (n = 1,935, 7.1%), which included acute bronchitis, pharyngitis, sinusitis and upper respiratory tract infections. The median total duration of therapy in patients who received antibiotics, including inpatient and after discharge, was 7 days (IQR 4-10).

Across all 111 sites, the median inpatient antibiotic DOT per 1,000 days present was 73.5 (IQR, 60.4–86.1) The most commonly prescribed agents, as quantified by DOT per 1,000 days-present, were trimethoprim-sulfamethoxazole (10.7), cephalosporins (9.5), tetracyclines (8.8), anti-influenza medications (8.3) and fluoroquinolones (8.0).

### Manual chart reviews to assess guideline-concordance

Table 2 shows the findings from the manual chart reviews, including the percentage of cases in which the reviewers disagreed. Among 111 UTI cases, 27 cases (24.3%) had documentation of altered mental status. For 56 cases (50.5%), documentation indicated the absence of local genitourinary symptoms, and an additional 7 cases (6.3%) had negative urine cultures collected before antibiotics were initiated. Therefore, the diagnosis of UTI was not supported by history or laboratory findings in 63 cases (56.8%). In 21 cases (18.9%) documentation could not be found that indicated that anyone had asked the patient about local genitourinary symptoms.

For 101 SSTI cases, a documented history of active intravenous drug use was present in 25 cases (24.8%). In 6 (5.9%) cases with chronic bilateral lower leg skin changes, we found no documented evidence of cellulitis but antibiotics had still been prescribed for these patients. In the remaining 95 cases with infection (39 purulent and 56 nonpurulent), antibiotic selection and duration was guideline concordant in 75 (78.9%) and 86 (90.5%) cases, respectively.

The 108 ARI cases included 55 cases (50.9%) of upper respiratory tract infections, 20 cases (18.5%) with pharyngitis, 13 cases (12.0%) of sinusitis, and 20 cases (18.5%) of acute bronchitis, which included 1 case recategorized as an acute exacerbation of chronic obstructive pulmonary disease. In 76 cases (70.4%) an antibiotic was not indicated and not prescribed. In 29 cases (26.9%) an antibiotic was not indicated but had still been prescribed, and in 3 cases (2.8%) an antibiotic was both indicated and prescribed.

Antibiotic recommendations were made by emergency department clinicians in 81 (25.3%) of the manually reviewed cases; recommendations were made by internal medicine clinicians in 89 cases (27.8%); and recommendations were made by infectious diseases clinicians in 2 cases (0.6%). At least 1 of these services was involved in 161 (50.3%) of all reviewed cases, including 54 cases (48.7%) with UTI, 70 cases (69.3%) with SSTI, and 37 cases (34.3%) with ARI. When these nonpsychiatric services were involved, we detected no differences in the quality of antibiotic-prescribing for UTIs or SSTIs and less guideline-concordant management for ARI (Supplementary Table 3 online).

## Discussion

In this study across 111 inpatient mental health units in the VHA, 1 of every 10 patients was exposed to an antibiotic. In contrast, studies in acute-care hospitals have reported that half of all patients are exposed to an antibiotic.^4^

The most common reason for antibiotic therapy in these inpatient mental health wards was a suspected UTI, but most UTI cases had the documented absence of local genitourinary symptoms or had no documentation suggesting that a clinician inquired about these symptoms. In many cases, the presence of altered mental status appeared to be a driver of antibiotic therapy even though antibiotics are not recommended for bacteriuria in the context of cognitive impairment alone.^5^ Overall, these findings suggest that UTI is commonly misdiagnosed and therefore represent an optimal target for stewardship efforts. We also identified some opportunities to improve antibiotic-prescribing for SSTIs and ARIs.

Our results revealed that emergency department and internal medicine clinicians commonly provided treatment recommendations in mental health patients with suspected infections. Because antibiotic-prescribing was not consistently better when these services were involved, antibiotic stewardship interventions to improve antibiotic prescribing in VHA mental health units must target both mental health clinicians as well as these other physician disciplines.^6^

Our findings are in line with prior work that has described the frequent misdiagnosis of UTIs.^7,8^ We speculate that a major contributor to the overdiagnosis of UTIs is the seemingly routine performance of urinalyses in admitted patients.^9^ More thoughtful ordering and interpretation of urine studies may help mitigate incorrect UTI diagnoses and subsequent unnecessary antibiotic use.^10^

Our study had a few limitations. First, our manual chart reviews were dependent on the quality of the clinicians’ documentation and our ability to ascertain key data elements in the medical record. To minimize the possibility of omission, we had 2 team members review each included case. Second, to assess guideline concordance, we sampled an equal number of cases from each facility regardless of bed size. As a result, antibiotic-prescribing practices at smaller facilities, which contributed less to total national antibiotic use, may have disproportionately influenced our overall assessments of guideline discordance. Third, because we specifically looked for cases coded as a UTI, we may have overlooked asymptomatic cases in which abnormal urine studies were correctly not attributed to a UTI. Finally, our findings may not be generalizable to non-VHA settings.

In conclusion, our study measured and evaluated antibiotic-prescribing across inpatient mental health units, which have been overlooked by both antibiotic stewardship metrics and by prior research. Future antibiotic stewardship interventions in mental health units should focus on the accurate diagnosis of UTIs while targeting the different physician disciplines involved in antibiotic decision making for mental health patients.

## Supplementary material

For supplementary material accompanying this paper visit https://doi.org/10.1017/ice.2021.432. click here to view supplementary material
