# Supplementary material for: Antibiotic prescribing in mental health units across the Veterans’ Health Administration: How much and how appropriate?
Source: Infect Control Hosp Epidemiol. 2021 Oct 21;44(2):308–11. doi: 10.1017/ice.2021.432 (PMC9929705; doi:10.1017/ice.2021.432)
Supplement: Supplementary file 1 [file S0899823X21004323sup001.pdf]

**Supplemental Table 1. ICD-10 codes that were used to identify eligible cases for manual chart reviews**

| Infection type | ICD-10 codes |
|---|---|
| **Urinary tract infections** | N30.90 (cystitis, unspecified without hematuria)<br>N30.91(cystitis, unspecified with hematuria)<br>N30.80(other cystitis without hematuria)<br>N30.81 (other cystitis with hematuria)<br>N30.01 (acute cystitis with hematuria)<br>N30.00 (acute cystitis without hematuria)<br>B37.41 (candida cystitis and urethritis)<br>N39.0(UTI, site not specified) |
| **Skin and soft tissue infection (limb only)** | L03.90 (cellulitis, unspecified)<br>L08.89, L08.9 (local infection of skin and subcutaneous tissue)<br>A46 (erysipelas)<br>L03.011, L03.012, L03.019 (cellulitis of fingers)<br>L03.021, L03.022, L03.029, L03.041, L03.042, L03.049 (acute lymphangitis of finger, toe, or unspecified finger/toe)<br>L03.031, L03.032, L03.039 (cellulitis of toe)<br>L03.113, L03.114, L03.115, L03.116, L03.119 (cellulitis of limb)<br>L03.121, L03.122, L03.123, L03.124, L03.125, L03.126, L03.129 (acute lymphangitis of limb)<br>L038.18 (cellulitis of other sites)<br>L03.90 (cellulitis unspecified)<br>L03.898, L03.91, L04.8, L04.9 (acute lymphangitis/lymphadenitis of other sites/unspecified)<br>L04.2, L04.3 (acute lymphangitis of upper/lower limbs)<br>L02.413, L02.414, L02.415, L02.416 (cutaneous abscess of limb)<br>L02.423, L02.424, L02.425, L02.426, L02,429 (furuncle of limb)<br>L02.433, L02.434, L02.435, L02.436, L02.439 (carbuncle of limb)<br>L02.511, L02.512, L02.519, L02.521, L02.522, L02.529, L02.531, L02.532, L02.539 (furuncle, carbuncle, cutaneous abscess of hand)<br>L02.611, L02.612, L02.619, L02.621, L02.622, L02.629, L02.631, L02.632, L02.639 (furuncle, carbuncle, cutaneous abscess of foot)<br>L02.818, L02.91 (cutaneous abscess at other sites/unspecified)<br>L02.828, L02.92, L02.838/L02.93 (carbuncle at other sites/unspecified) |
| **Acute respiratory infections** | J20.0, J20.1, J20.2, J20.3, J20.4, J20.5, J20.6, J20.7, J20.8, J20.9, J22, J40, J21.0, J21.1, J21.8, J21.9 (acute bronchitis)<br>J01.00, J01.01, J01.10, J01.11, J01.20, J01.21, J01.30, J01.31, J01.40, J01.41, J01.80, J01.81, J01.90, J01.91 (acute sinusitis)<br>J02.0, J02.8, J02.9, J03.00, J03.01, J03.80, J03.81, J03.90, J03.91 (pharyngitis)<br>J06.0, J06.9, J00., J04.0, J04.2, J05.0 (upper respiratory tract infection) |

**Supplemental Table 2. Reasons for excluding cases with eligible ICD codes from the manual chart reviews**

| Infection type | Reason for exclusion | Number of cases that met exclusion criterion |
|---|---|---|
| **Urinary tract infections** | Initiated antibiotic therapy at an outside facility but documentation of prior antibiotic therapy was not complete | 10 |
| | Antibiotic therapy was never initiated | 8 |
| | Indwelling urinary catheter present | 5 |
| | Chart documentation did not indicate that clinicians were actively considering a UTI | 4 |
| | Patient was transferred to an acute-care ward | 2 |
| | A second infection was present | 2 |
| | A urinalysis was never performed | 1 |
| **Skin and soft tissue infections** | Chart documentation was incomplete and/or did not indicate that clinicians thought cellulitis was an active problem | 51 |
| | Cellulitis of face, torso, abdominal wall, perianal region or surgical site | 36 |
| | Complicated infection, including chronic wound, water exposure, animal/human bite, necrotizing infection, osteomyelitis/tenosynovitis | 15 |
| | Initiated antibiotic therapy at an outside facility but documentation of prior antibiotic therapy was not complete | 9 |
| **Acute respiratory infections** | Chart documentation was incomplete | 21 |
| | Diagnosis of pneumonia | 5 |
| | Initiated antibiotic therapy at an outside facility but documentation of prior antibiotic therapy was not complete | 3 |
| | Chart documentation did not indicate that clinicians were actively considering an ARI | 2 |
| | A second infection was present | 1 |

**Supplemental Table 3. The association between selected aspects of guideline-concordant diagnosis/management and consultation with Emergency Medicine or Internal Medicine clinicians**[1]

| Infection type | Total n (%) | Any consultation n (%) | No consultation n (%) | p-value[2] |
|---|---|---|---|---|
| **UTI (N=111)** | | | | |
| Supported diagnosis | 27 (24.3) | 15 (27.8) | 12 (21.1) | 0.41 |
| Unsupported diagnosis | 63 (56.8) | 30 (55.6) | 33 (57.9) | |
| Unable to assess | 21 (18.9) | 9 (16.7) | 12 (21.1) | |
| **SSTI (N=95)** | | | | |
| Guideline-concordant antibiotic selection | 75 (78.9) | 56 (83.6) | 19 (67.9) | 0.09 |
| Guideline-discordant antibiotic selection | 20 (21.1) | 11 (16.4) | 9 (32.1) | |
| **ARI (N=108)** | | | | |
| Guideline-concordant management[3] | 79 (73.1) | 20 (54.1) | 59 (83.1) | <0.01 |
| Guideline-discordant management | 29 (26.9) | 17 (45.9) | 12 (16.9) | |

Abbreviations: ARI acute respiratory tract infection; SSTI skin and soft tissue infection; UTI urinary tract infection

1. Emergency Department clinicians were involved in the antibiotic decision-making for 81 cases, and Internal Medicine clinicians were involved in 89 cases. In 2 cases, the Infectious Disease service was consulted; these 2 cases are also included in this table.
2. The p-value reflects the results of a chi-square test. For UTI, the test compared the frequency of having a supported diagnosis in cases with and without a consultation. For SSTI and ARI, the test compared the frequency of guideline-concordant antibiotic selection in cases with and without a consultation.
3. Guideline-concordant management for ARIs was defined as prescribing an antibiotic when indicated or not prescribing an antibiotic when not indicated.

**Supplemental figure 1. Algorithm for assessing guideline-concordant management of cystitis**

**Cystitis**

**Exclusion criteria:**

- Pregnancy
- Died during hospital stay
- Antibiotic stopped on or before day 3 of antibiotic therapy
- ICD code for cystitis does not match stated indication
- Transferred to or from VA and antimicrobial therapy before or after transfer is not documented
- 2nd infection concomitant to cystitis
- Complicated urinary tract infection, such as prostatitis, pyelonephritis, or epididymo-orchitis
- No urinalysis results are available
- Indwelling urinary catheter present, including urethral or suprapubic catheter

Yes → Excluded from review

No ↓

**Did the urinalysis show ≥ 5 white blood cells per high-power field?**

No → **UTI was not present**

Yes ↓

**Was the urine culture, obtained prior to antibiotics, negative or does the result reflect skin contamination?**

Yes → **UTI was not present**

No ↓

**Does patient report having the acute onset of at least one of the following symptoms?**

- Dysuria
- Urinary urgency
- Urinary frequency
- Suprapubic pain
- Hematuria

No → **UTI was not present**

Un-known → **Choose "Unable to assess, because GU symptoms were not documented" and use below criteria to evaluate appropriateness**

Yes ↓

**Appropriateness criteria assessed on day 3 of antibiotics**
1) changing to or continuing an active oral/IM agent based on confirmed susceptibilities of the isolated pathogen;
2) changing from an inactive agent to an active oral/IM agent, based on confirmed susceptibilities of the isolated pathogen;
3) in the absence of a urine culture, continuing an appropriate empiric antibiotic agent (assuming the patient is demonstrating a clinical response): amoxicillin/clavulanate, cefpodoxime, cefuroxime, cephalexin, ciprofloxacin, fosfomycin, levofloxacin, nitrofurantoin, and trimethoprim/sulfamethoxazole

Appropriate antibiotic duration was 7 days except in cases of uncomplicated cystitis, defined as a lower urinary tract infection in a healthy, non-pregnant, pre-menopausal woman with no history of an abnormal urinary tract. In these cases of uncomplicated cystitis, only shorter antibiotic courses were considered appropriate (3 days for TMP/SMX and ciprofloxacin; 5 days for nitrofurantoin and oral beta-lactams).

References: (1) Gupta K, Hooton TM, Naber KG, et al. International Clinical Practice Guidelines for the Treatment of Acute Uncomplicated Cystitis and Pyelonephritis in Women. *Clin Infect Dis* 2011; 52: e103-e120. (2) Griebel ME, Heintz, Alexander B, Egge J, Goto M, Livorsi DJ. Understanding changes in the standardized antimicrobial administration ratio for total antimicrobial use after implementation of prospective audit and feedback. *Infect Control Hosp Epidemiol* 2018;39: 1476-1479.

## Supplemental figure 2. Algorithm for assessing guideline-concordant management of cellulitis

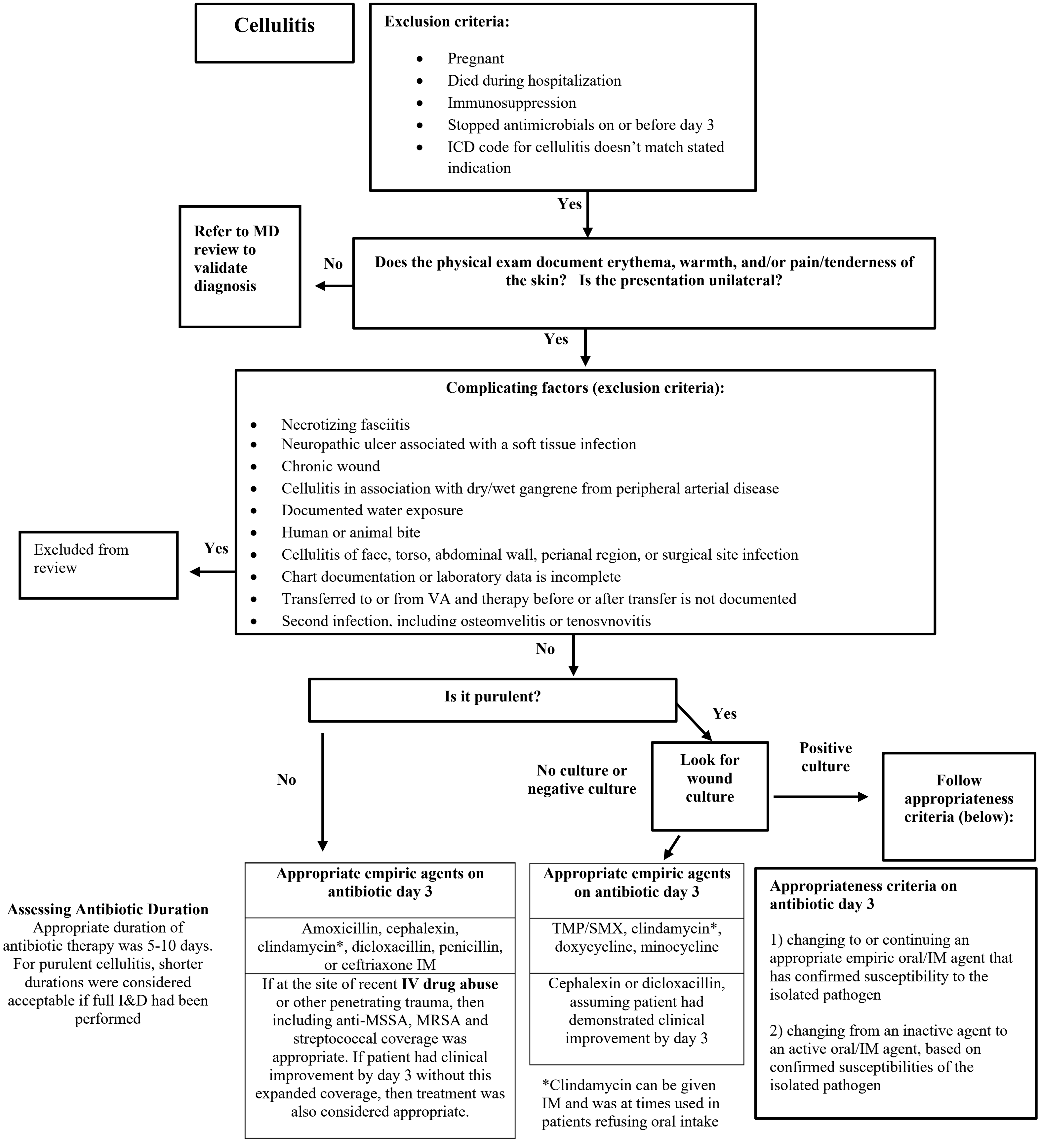


References: (1) Stevens DL, Bisno AL, Chambers HF, et al. Practice guidelines for the diagnosis and management of skin and soft tissue infections: 2014 update by IDSA. Clin Infect Dis 2014; 59: e10-52. (2) Griebel ME, Heintz, Alexander B, Egge J, Goto M, Livorsi DJ. Understanding changes in the standardized antimicrobial administration ratio for total antimicrobial use after implementation of prospective audit and feedback. *Infect Control Hosp Epidemiol* 2018;39: 1476-1479.

**Supplemental figure 3. Algorithm for assessing guideline-concordant management of acute bronchitis**

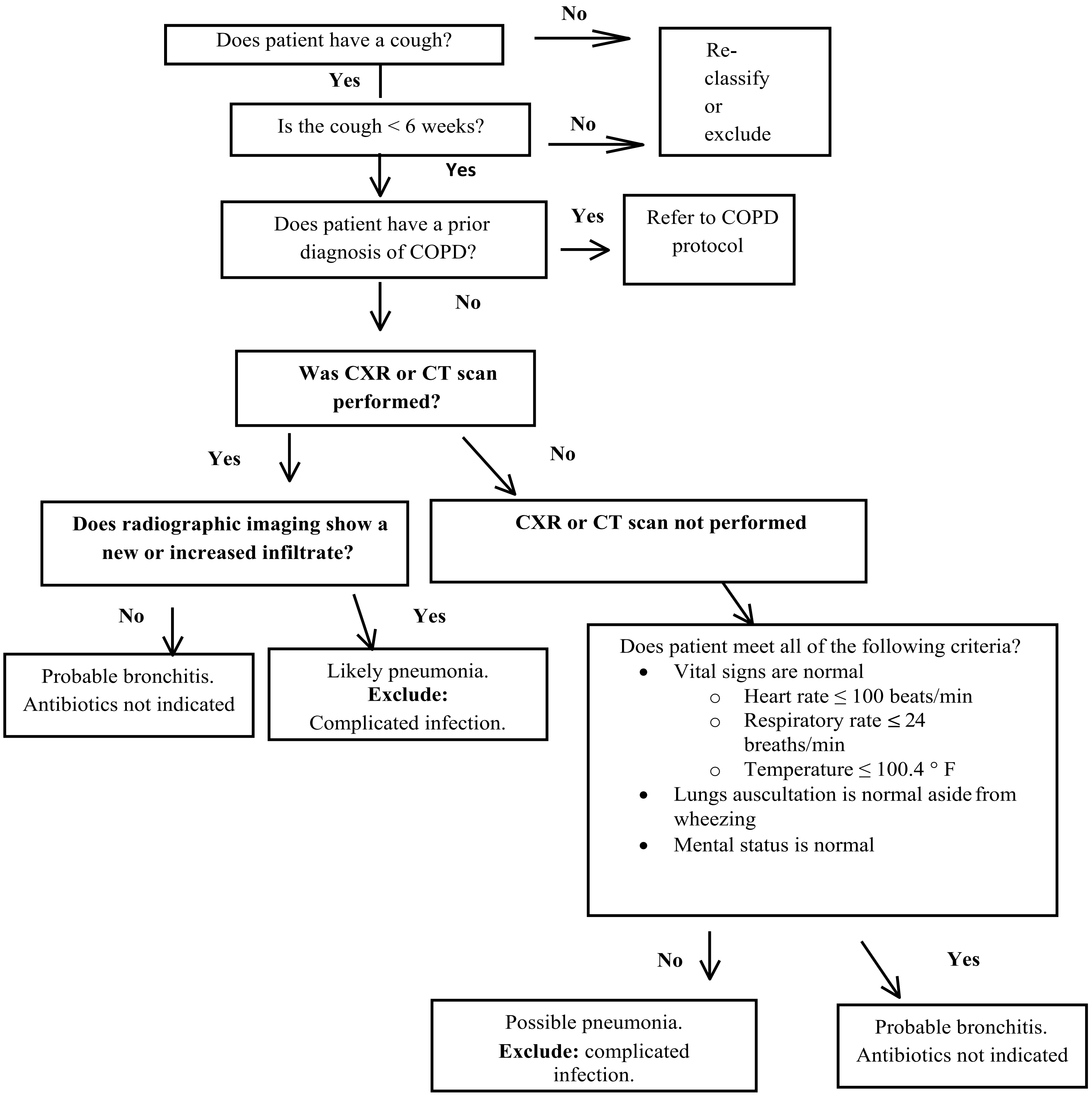


Irwin RS, Baumann MH, Bolser DC, et al. Diagnosis and management of cough: ACCP evidence-based clinical practice guidelines. *Chest*. 2006;129(1 Suppl).

Harris AM, Hicks LA, Qaseem A, et al.. Appropriate Antibiotic Use for Acute Respiratory Tract Infection in Adults: Advice for High-Value Care From the American College of Physicians and the Centers for Disease Control and Prevention. *Ann Intern Med.* Mar 15 2016;164(6):425-434.

**Supplemental figure 4. Algorithm for assessing guideline-concordant management of acute exacerbations of chronic obstructive pulmonary disease (COPD)**

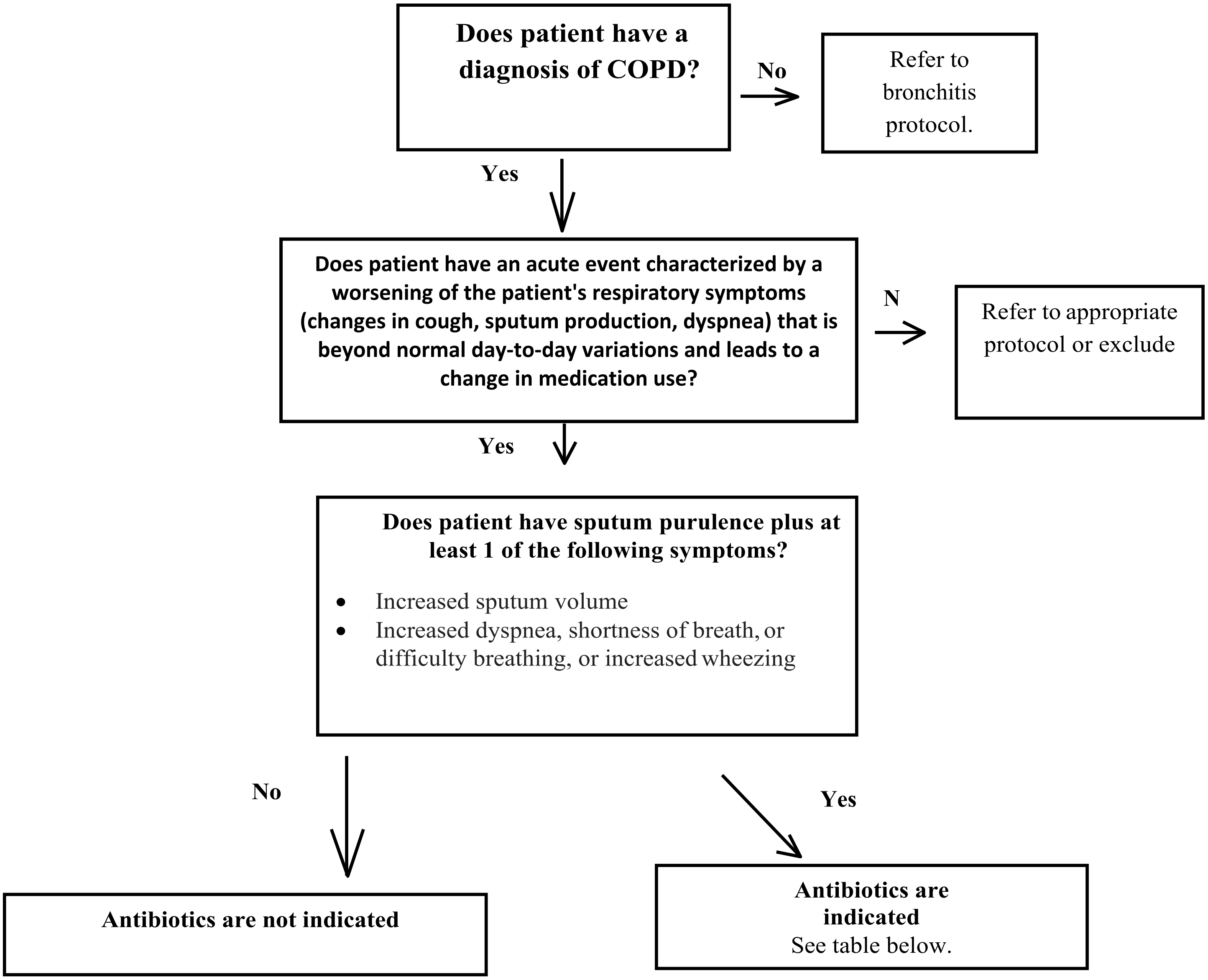


| Drug | Dose | Duration |
|---|---|---|
| Amoxicillin/clavulanate | 500 mg/125 mg TID or 875 mg/125 mg BID | 5-7 days |
| Azithromycin | 500 mg x1, 250 mg daily | 5-7 days |
| Cefuroxime | 250-500 mg BID | 5-7 days |
| Cefdinir | 300 mg BID or 600 mg daily | 5-7 days |
| Cefpodoxime | 200 mg BID | 5-7 days |
| Doxycycline | 100 mg BID | 5-7 days |

Global Initiative for Chronic Obstructive Lung Disease. Global Strategy for the Diagnosis, Management, and Prevention of Chronic Obstructive Pulmonary Disease (2020 report). https://goldcopd.org/wp-content/uploads/2019/12/GOLD-2020-FINAL-ver1.2-03Dec19_WMV.pdf. Accessed August 24, 2021.

**Supplemental figure 5. Algorithm for assessing guideline-concordant management of pharyngitis**

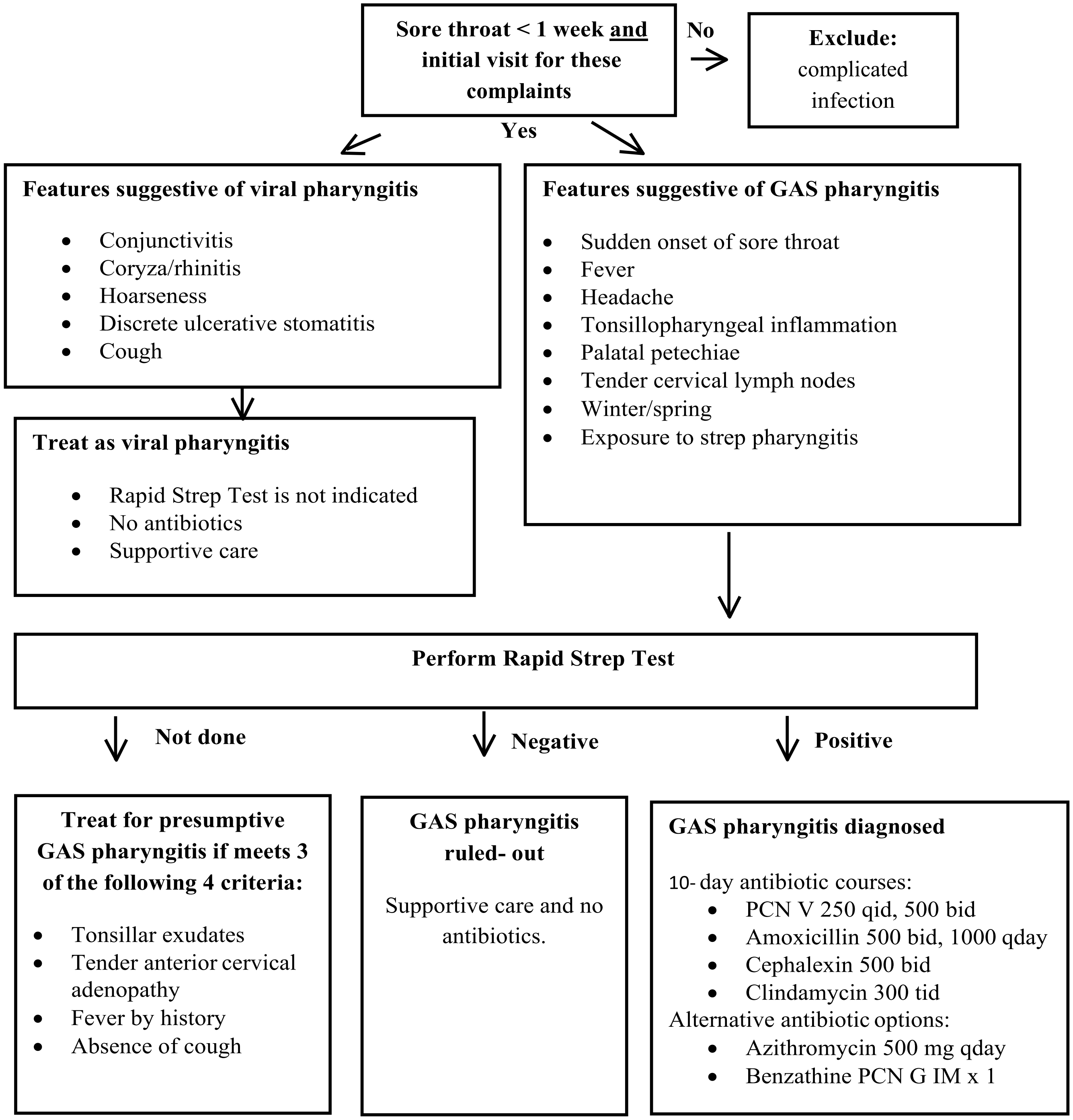


Harris AM, Hicks LA, Qaseem A, et al.. Appropriate Antibiotic Use for Acute Respiratory Tract Infection in Adults: Advice for High-Value Care From the American College of Physicians and the Centers for Disease Control and Prevention. *Ann Intern Med.* Mar 15 2016;164(6):425-434.

European Society for Clinical Microbiology and Infectious Diseases Sore Throat Guideline Group. Guideline for the management of acute sore throat. Clin Microbiol Infect 2012; 18 (Suppl. 1): 1-27.

**Supplemental figure 6. Algorithm for assessing guideline-concordant management of acute sinusitis**

**Has patient been ill for < 4 weeks?**

No → **Exclude:** Complicated infection

Yes ↓

**Does patient have at least 2 major or 1 major and ≥2 minor symptoms?**

No → Re-classify or exclude

Yes ↓

| Major | Minor Criteria |
|---|---|
| Purulent anterior nasal discharge or purulent or discolored posterior nasal discharge | Headache |
| Nasal congestion or obstruction | Ear pain, pressure or fullness |
| Fever (acute sinusitis) | Halitosis |
| Facial congestion/fullness or facial pain/pressure | Dental pain |
| Hyposmia or anosmia | Cough or fatigue |
| | |

**Does patient meet one of the following criteria?**

1. Onset with persistent symptoms that last ≥ 10 days and are not improving
2. Onset with severe symptoms, characterized by high fever of at least 39°C (102°F) and purulent nasal discharge for at least 3 consecutive days at the beginning of illness
3. Onset with worsening symptoms (new onset fever, headache, nasal discharge), characterized by typical viral URI symptoms that appear to improve followed by the sudden onset of worsening symptoms after 5–6 days (''double-sickening'')

No ↓ **Antibiotics not indicated**

Yes ↓ **Antibiotics are indicated**

| **No penicillin allergy** | | |
|---|---|---|
| **Drug** | **Dose** | **Duration** |
| Amoxicillin-clavulanate | 500 mg/125 mg TID or<br>875 mg/125 mg BID/TID | 5-7 days |
| Cefdinir | 300 mg BID | 5-7 days |
| Doxycycline | 100 mg BID or 200 mg daily | 5-7 days |

| **Penicillin allergy** | | |
|---|---|---|
| **Drug** | **Dose** | **Duration** |
| Doxycycline | 100 mg BID or 200 mg daily | 5-7 days |
| Levofloxacin | 500 – 750 mg daily | 5-7 days |
| Moxifloxacin | 400 mg daily | 5-7 days |
| Cefdinir | 300 mg BID OR 600 mg daily | 5-7 days |

Harris AM, Hicks LA, Qaseem A, et al.. Appropriate Antibiotic Use for Acute Respiratory Tract Infection in Adults: Advice for High-Value Care From the American College of Physicians and the Centers for Disease Control and Prevention. *Ann Intern Med.* Mar 15 2016;164(6):425-434.

**Supplemental figure 7. Algorithm for assessing guideline-concordant management of upper respiratory tract infections, not otherwise specified**

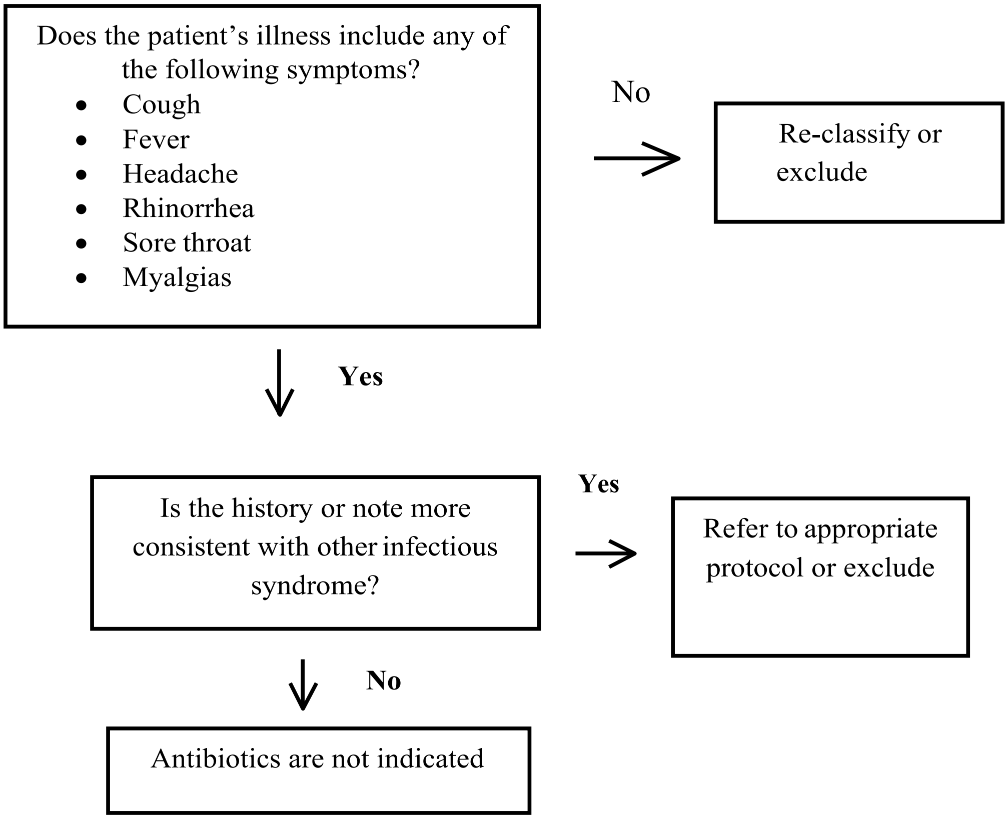



Harris AM, Hicks LA, Qaseem A, et al.. Appropriate Antibiotic Use for Acute Respiratory Tract Infection in Adults: Advice for High- Value Care From the American College of Physicians and the Centers for Disease Control and Prevention. *Ann Intern Med.* Mar 15 2016;164(6):425-434.
